# DETECTING NARCISSISM FROM DAILY LANGUAGE USE: A MACHINE LEARNING APPROACH

**DOI:** 10.1093/geroni/igac059.2278

**Published:** 2022-12-20

**Authors:** Shiyang Zhang, Sibo Gao, Karen Fingerman

**Affiliations:** University of Texas at Austin, Austin, Texas, United States; School of Human Ecology, Austin, Texas, United States; The University of Texas at Austin, Austin, Texas, United States

## Abstract

High-quality social contacts are important for older adult’s emotional well-being whereas narcissism, characterized by self-centeredness, may undermine the quality of personal relationships. Narcissism may be associated with poorer quality relationships because narcissistic adults are more self-centered in their daily social interactions. The current study examined the associations between linguistic features of conversation throughout the day and narcissism using a machine learning approach. Older adults aged 65–89 (N = 281, Mage = 74.07) wore an unobtrusive electronically activated recorder (EAR) throughout the day for 4 to 6 days. The EAR was activated 30 seconds every 7 minutes to capture conversations occurring in a natural setting. The sound files that contained participant’s speech (N = 28,243) were transcribed verbatim. We used Linguistic Inquiry and Word Count (LIWC) to extract linguistic features from the transcriptions (nfeature = 81; e.g., function words, affective processes). Linguistic features were analyzed using random forest algorithm which is a supervised machine learning method that can evaluate features’ performances in predicting older adult’s narcissism. The model reached an accuracy of 62% and results showed that total word count, more auxiliary verbs (e.g., will), more swear words (e.g., damn), and less assent (e.g., agree) were the most powerful predictors of narcissism. Drawing on sound files collected from real-life interactions, findings indicate higher usage of aggressive and disagreeable words among more narcissistic individuals, which probably leads to poorer quality relationships with social partners. Interventions aiming to improve contact quality and emotional well-being may consider individuals’ word use in daily interactions.

